# Toward the use of mixed microbial cultures for the biological production of adipic and levulinic acid

**DOI:** 10.3389/fmicb.2023.1224543

**Published:** 2023-06-28

**Authors:** Fernanda Pinto-Ibieta, Mara Cea, Antonio Serrano, Fernando E. Felissia, María Cristina Area, Francisco Cabrera, Gustavo Ciudad

**Affiliations:** ^1^Departamento de Procesos Industriales, Facultad de Ingeniería, Universidad Católica de Temuco, Temuco, Chile; ^2^Departamento de Ingeniería Química, Universidad de La Frontera, Temuco, Chile; ^3^Scientific and Technological Bioresource Nucleus (BIOREN), Universidad de La Frontera, Temuco, Chile; ^4^Institute of Water Research, University of Granada, Granada, Spain; ^5^Department of Microbiology, Pharmacy Faculty, University of Granada, Granada, Spain; ^6^IMAM, UNaM, CONICET, FCEQYN, Programa de Celulosa y Papel (PROCYP), Posadas, Argentina; ^7^Instituto de Ciencias Químicas Aplicadas, Universidad Autónoma de Chile, Temuco, Chile; ^8^Instituto del Medio Ambiente (IMA), Universidad de La Frontera, Temuco, Chile

**Keywords:** adipic acid, levulinic acid, mixed microbial cultures (MMC), hemicellulose hydrolysate, feast and famine

## Abstract

Biological synthesis of high added-value compounds like adipic acid (AA), levulinic acid (LA), or polyhydroxybutyrate (PHB) using pure culture has been separately reported. However, pure culture requires sterile conditions and the use of specific carbon sources resulting in high operating costs. Different alternatives based on the use of mixed microbial cultures (MMC) have been explored to resolve this problem. MMC have been widely reported for the production of PHB, but scarcely reported for LA production and never for AA synthesis. This work presents a novel strategy for the co-production of AA LA, and PHB using MMC. The strategy consists in selecting an MMC producer of AA, LA and PHB from an inoculum obtained from a wastewater treatment plant, which is then subjected to the feast and famine culture strategy in a sequential batch reactor, coupled with a batch reactor step to enhance the accumulation of AA and LA. The results showed that the MMC could produce a 16 ± 2, 23 ± 1 and 5 ± %1 (g compound/g volatile solids) of AA, LA and PHB, respectively, using a non-fermented residual biomass rich in pentose, namely synthetic hemicellulose hydrolysate (SHH) as the carbon source. These results contribute to generating future research to better understand and optimise the biosynthesis of these compounds by MMC.

## 1. Introduction

The biological process to produce high value-added compounds has been gaining interest, as these processes form part of the efforts to move from a fossil fuel economy to one based on renewable resources (Jeong et al., 2018). Some of these high value-added compounds that can be produced using biological processes are adipic acid (AA), levulinic acid (LA), and polyhydroxybutyrate (PHB) (Rodriguez-Perez et al., 2018; Pinto-Ibieta et al., 2020, 2021). LA is a platform molecule that allows the production of a wide range of compounds applied in the chemical, food, and agrochemical industries, being considered among the 12 molecules with the highest added value in the world (Lappalainen and Dong, 2019; Morakile et al., 2022; Ukawa-Sato et al., 2023). AA is use to produce of nylon 6.6, a polymer in very high demand in the textile and automotive industries (Riveiro et al., 2020; Wu et al., 2023). It can also be used as an intermediate to produce different compounds like cyclopentanone and 1.6-hexanediol, which are important in the fragrance and resin production industries, respectively (Corona et al., 2018; Kruyer et al., 2020). Finally, PHB are biodegradable polyesters that can be produced in bioprocesses from renewable resources in contrast to fossil-based bio-recalcitrant polymers (Cassuriaga et al., 2018; Correa-Galeote et al., 2022a). Biological synthesis of AA, LA, and PHB using pure culture has been reported separately. For instance, engineered bacterial strains like *Saccharomyces cerevisiae* and *Pseudomonas putida* are able to synthesise AA from different substrates, even hexoses and lignin (Raj et al., 2018; Zhao et al., 2018; Wu et al., 2023). LA synthesis can be carried out by fermentation of pentoses using engineered *Saccharomyces cerevisiae* and *Pichia stipis* (Zhangelinni, 2017). PHB production from pure culture using pentoses and hexoses has been widely recognised and exploited (Li and Wilkins, 2020). In contrast with pure culture, the use of mixed microbial cultures (MMC) is gaining attention. MMC involve lower operating costs and can be adapted to the use of agro-industrial waste as a carbon source, minimising both the operational cost and the ecological footprint of these processes (Huang et al., 2018; Mohamad Fauzi et al., 2019; Yin et al., 2019a; Correa-Galeote et al., 2022b). The use of MMC subjected to a feast and famine strategy is mainly focused on PHB production using volatile fatty acids as the carbon source (Rodriguez-Perez et al., 2018). Less reported, however, has been the use of non-fermented waste as a carbon source for PHB production (Pinto-Ibieta et al., 2020, 2021). Indeed, the ability of the MMC to produce PHB when the carbon source used was reduction sugars obtained from lignocellulosic hydrolysates has been described (Yin et al., 2019b, 2020). However, in addition to PHB production, it seems that non-fermented waste as feed for MMC could favour the accumulation of other added-value compounds by controlling operational parameters such as the dissolved oxygen, the organic loading rate or the feast/famine cycle length (Pinto-Ibieta et al., 2021). For example, Pinto-Ibieta et al. (2020) reached a LA production of up to 32% (g compound/g *VS*) (*VS*, volatile solids) with a MMC fed with pentose-rich hemicellulose hydrolysate. Meanwhile, Argiz et al. (2021) managed to accumulate 18% (g compound/g *VS*) of triglycerides and 26% (g compound/g *VS*) of PHA when the MMC were fed with residual fish-canning oil. The co-production of some compounds could be possible because of a close relationship between metabolic routes. For example, the intracellular production of both AA and LA involve the pentose phosphate pathway by the transformation of the pentoses to pyruvic acid and its subsequent oxidation to acetyl-CoA (Lee et al., 2019; Miscevic et al., 2019). The synthesis of LA or AA is then carried out from succinyl CoA after the acetyl-CoA enters the Krebs cycle (Lee et al., 2019). Although the metabolic route for AA accumulation is known, to the best of our knowledge, the ability to produce AA by MMC has not been previously reported.

Therefore, this study is focused mainly on (1) proving, if possible, the use of MMC to produce AA through the use of non-fermented feedstock rich in xylose as the carbon source, and (2) if possible, co-producing AA, with LA or PHB using the MMC. Thus, the main novelty of this work lies in opening up a new strategy for the biological synthesis of AA using MMC in co-production with LA and PHB.

## 2. Materials and method

### 2.1. Operation of SBRs to adapt the MMC

Six 2-L sequential batch reactors (SBR) were simultaneously operated to evaluate the adaptation of an MMC to synthesise AA in co-production with LA under a feast and famine regime. The first condition (SBR1), assayed in triplicate, was fed with synthetic hemicellulose hydrolysate, where the composition in percentage (% g compound/g *VS*) was a residual stream of lignocellulosic process fractionation (Vallejos et al., 2017): 78% xylose, 9.0% acetic acid, 5.06% furfural, 4.7% arabinose, 2.3% glucose, 0.8% cellobiose, and 0.14% hydroxymethylfurfural. A second condition (SBR2), assayed in triplicate, was operated using acetic acid as the only carbon source. Both substrates were selected as models for comparing the use of non-fermented (SHH) versus acetate substrate as the carbon source to produce high added-value compounds (AA and LA) instead of PHB when the MMC are subjected to a feast and famine regimen. SBR1 and SBR2 were supplemented with the nutrients and minerals necessary for bacterial growth, including: 132 mg of peptone/L, 68 mg of beef extract/L, 112 mg of (NH_4_)_2_SO_4_/L, 49 mg of KH_2_PO_4_, 66 MgSO_4_/L, 170 mg of NH_4_Cl/L, 92 mg of K_2_HPO_4_/L, 45 mg KH_2_PO_4_/L, 600 mg MgSO_4_/L, 100 mg EDTA/L, 70 mg CaCl_2_/L, and 2 mL of a trace element solution (Huang et al., 2017). 10 mg Thiourea/L was also added to prevent nitrification. The trace elements solution was composed of 1,000 mg FeSO_4_·6H_2_O/L, 150 mg H_3_BO_3_/L, 150 mg CoCl_2_·6H_2_O/L, 120 mg MnCl_2_·4H_2_O/L, 120 mg ZnSO_4_·7H_2_O/L, 60 mg Na_2_MoO_4_·2H_2_O/L, 30 mg KI/L, and 30 mg CuSO_4_·5H_2_O/L.

SBR1 and SBR2 were inoculated with activated sludge from the municipal wastewater treatment plant in Temuco, Chile. Both reactors were operated under the same conditions: 4 L/min airflow, 30°C, 60 rpm, and a C/N/P ratio of 30/0.9/0.1 (in mmol/L). The strategy applied was the alternation of F/F periods through SBR operation to obtain MMC enriched with microorganisms capable of producing high added-value compounds. Two cycles per day were carried out in each SBR until a stable operation was reached. One cycle consisted of: a feed period (6 min) followed by an aerobic reaction (704 min) and 10 min of withdrawal (Huang et al., 2017; Wang et al., 2017). No settling phase was performed, and all excess biomass was withdrawn with the mixed liquor (Dionisi et al., 2005; Cabrera et al., 2019). Thus, the biomass retention time was equal to the hydraulic retention time. 0.25 L of substrate was fed into each reactor in each cycle using organic loading rate (OLR) of 60 Cmmol/L*day. At the end of each cycle, 0.25 L of the liquid was withdrawn by digital peristaltic pumps using a Compact DAQ system (cDAQ-9,178 chassis, National Instruments, Austin, TX, United States), with a routine programmed using the LabView software (National Instruments). SBR1 and SBR2 were operated for 100 consecutive cycles (50 days) since stable operation was reached from cycle 80 in a previous study (Pinto-Ibieta et al., 2020). To monitor the evolution of AA, LA, PHB, and biomass growth in both SBR, samples were taken at the end of a cycle once a week. Once a stable operation was determined, intermediate samples were taken during feeding cycles 80, 84, 88, and 90 from both reactors. The samples were taken in triplicate every hour to monitor the evolution of AA, AL, PHB, biomass, and carbon source uptake (pentoses and acetate) as a function of the time in the cycle. The mean and standard deviation of these cycles were calculated to corroborate the stability of the operation.

### 2.2. Batch assays to maximise the accumulation of target compounds

The MMC obtained from the operation of SBR1 and SBR2 were cultivated separately in batch reactors to evaluate their adaptability to the use of SHH and maximise their ability to produce LA, AA and/or PHB in a batch stage, regardless of the substrate used during the selection phase. SHH (described in point 2.1) was the only carbon source used in these batch assays. The maximum accumulation capacity of compounds was assayed without the addition of nitrogen, since it has been reported that restricting this nutrient in the culture could lead to the accumulation of storage compounds by the cell (Guerfali et al., 2018; Chong et al., 2019). Six assays, in triplicate, were carried out to evaluate the effect of increasing SHH doses (30, 75, and 120 Cmmol/L) on the production of the compounds of interest (Table 1). 80 mL of adapted MMC (with a concentration of 3 mg *VS*/L), obtained from each SBR reactor described in Section 2.1 were used as inoculum in Erlenmeyer flasks containing 120 mL of mineral medium without a nitrogen source (see Section 2.1), incubated for 24 h at 150 rpm and 30°C. ANOVA tests were performed to evaluate the significance of the differences observed for AA and LA accumulations at the different experimental conditions.

**Table 1:** Assays in batch reactors for the improvement of levulinic acid (LA), adipic acid (AA), and polyhydroxybutyrate (PHB) accumulation.

Batch No	SHH concentration (Cmmol/L)	Inoculum
1	120	MMC from SBR1
2	75	MMC from SBR1
3	30	MMC from SBR1
4	120	MMC from SBR2
5	75	MMC from SBR2
6	30	MMC from SBR2

SHH, synthetic hemicellulose hydrolysate; MMC, mixed microbial culture; SBR, sequential batch reactor.

### 2.3. Analytical methods

LA, AA, and PHB were quantified and identified according to Lappalainen and Dong (2019) and Serafim et al. (2004). A GC/MS Clarus 600 (Perkin Elmer) with an ELITE 1701 column 30 m × 0.25 mm × 0.25 um was used for identification, and a GC/FID Clarus 600 (Perkin Elmer) with CAPILAR NUKOLTM SUPELCO 30 m × 0.25 mm × 0.25 um for quantification. The calibration curve was obtained by injecting a series of standards at different concentrations of AA, LA, and PHB (Sigma Aldrich). The substrate consumption was measured by determining the concentration of reducing sugars and acetate in filtered samples (0.22 μm pore size PVDF membrane, Merck). To quantify the total reducing sugars, the dinitrosalicylic acid (DNS) reagent method was used (Zhang et al., 2012; Prasertsung et al., 2017). Acetate was determined by gas chromatography in a flame ionisation detector (Clarus 400, PerkinElmer), using a NukolTM capillary column (Sigma-Aldrich, Darmstadt, Germany). *VS* and soluble chemical oxygen demand (SCOD) were quantified using a standard technique (AWWA, 2005).

## 3. Results

### 3.1. Mixed microbial culture selection capable of producing LA, AA and PHB

#### 3.1.1. LA, AA and PHB concentration evolution over operation time of selection reactors using SHH and acetate

**Figure 1:**
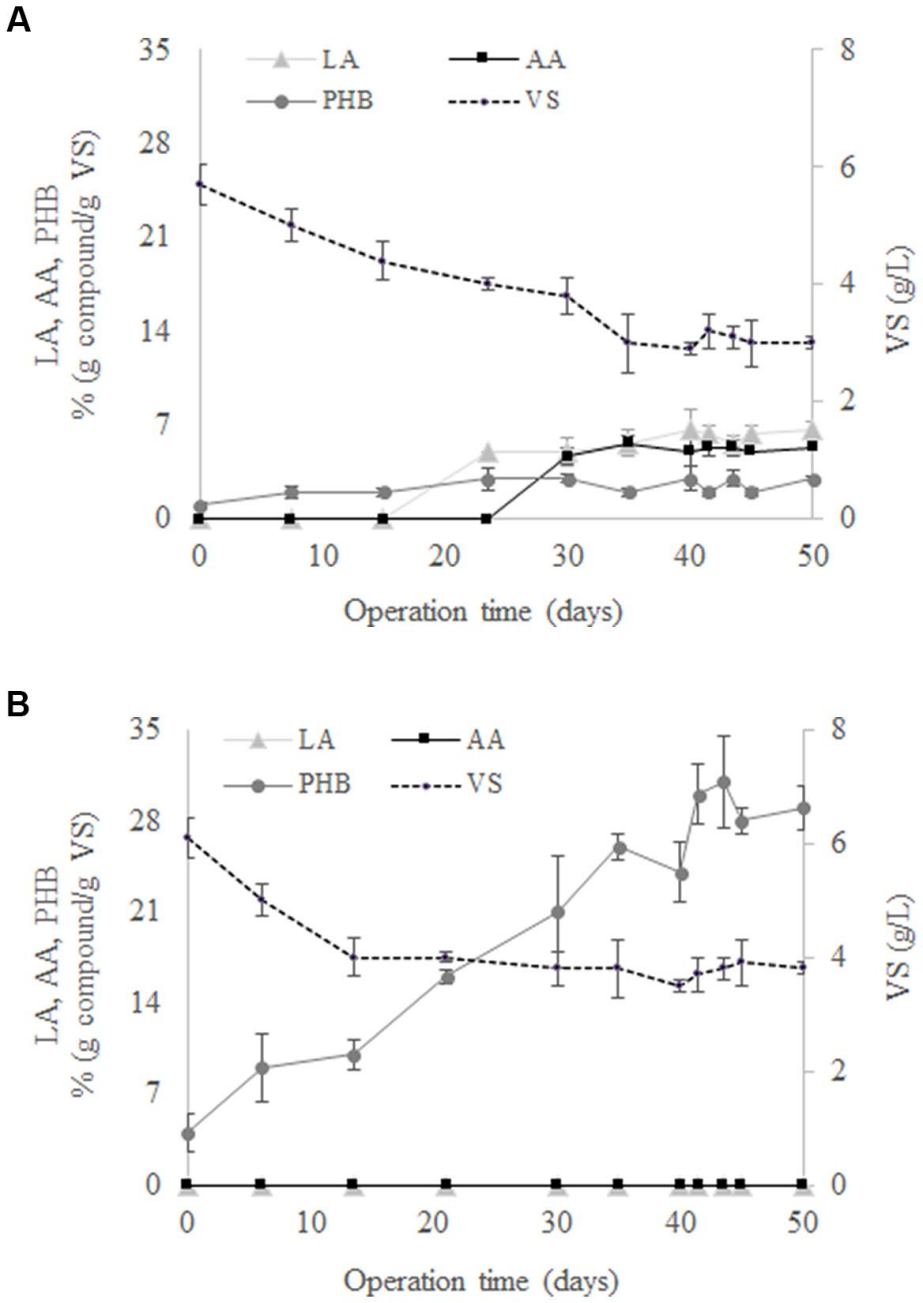
Evolution of levulinic acid (LA), adipic acid (AA), polyhydroxybutyrate (PHB), and volatile solids (*VS*) in the 50 days performed by SBR1 **(A)** and SBR2 **(B)**.

The evolution of LA, AA, PHB, and *VS* during the operation of SBR1 is shown in Figure 1A. An adaptation stage was observed during the first 30 days of operation followed by stable operation, reflected by constant concentrations of LA, AA, PHB and *VS* in successive cycle ends. At the same time, cell wash-out was observed since there was a gradual reduction in *VS* from 5.7 ± 0.3 g/L to 3 ± 0.1 g/L from days 0 to 35. According to Argiz et al. (2021), the decrease in the biomass concentration can be explained by the culture selection, with only the microbial populations capable of surviving at the F/F culture strategy remaining and synthesising SHH as a carbon source. The results showed that there was no AA and LA production at the beginning of the culture (Figure 1A). These compounds were produced after 20 days of operation, proving that the use of the F/F culture strategy was able to select a MMC that favours LA and AA production from SHH. Specifically, LA and AA reached mean concentration values of 5.9 ± 0.7% (g compound/g *VS*) and 5.2 ± 0.2% (g compound/g *VS*), respectively, from days 20 to 50 of operation (Figure 1A). On the other hand, the MMC selection for PHB production was not observed using SHH, since the PHB concentration stays constant throughout the operation time, i.e., 2.4 ± 0.5% (g compound/g *VS*).

Figure 1B shows that, contrary to the findings with SBR1, AA and LA were not synthesised when acetate was used as the carbon source in SBR2. Under these conditions, only PHB was determined in the reactor throughout the experimental time. In particular, PHB reached a mean value of 29.5 ± 1.3% (g compound/g *VS*) once the steady state was reached during days 41–50 (Figure 1B). This behaviour was expected, since it is widely reported that MMC can transform volatile fatty acids into PHB (Lee et al., 2015; Pinto-Ibieta et al., 2021; Argiz et al., 2022). Therefore, the production of AA and LA was only possible when the conditions were against the PHB through the feeding of a complex carbon source like SHH.

#### 3.1.2. Analysis of the behaviour of LA, AA, PHB, acetate and pentoses inside the culture cycles of the selection reactors

**Figure 2:**
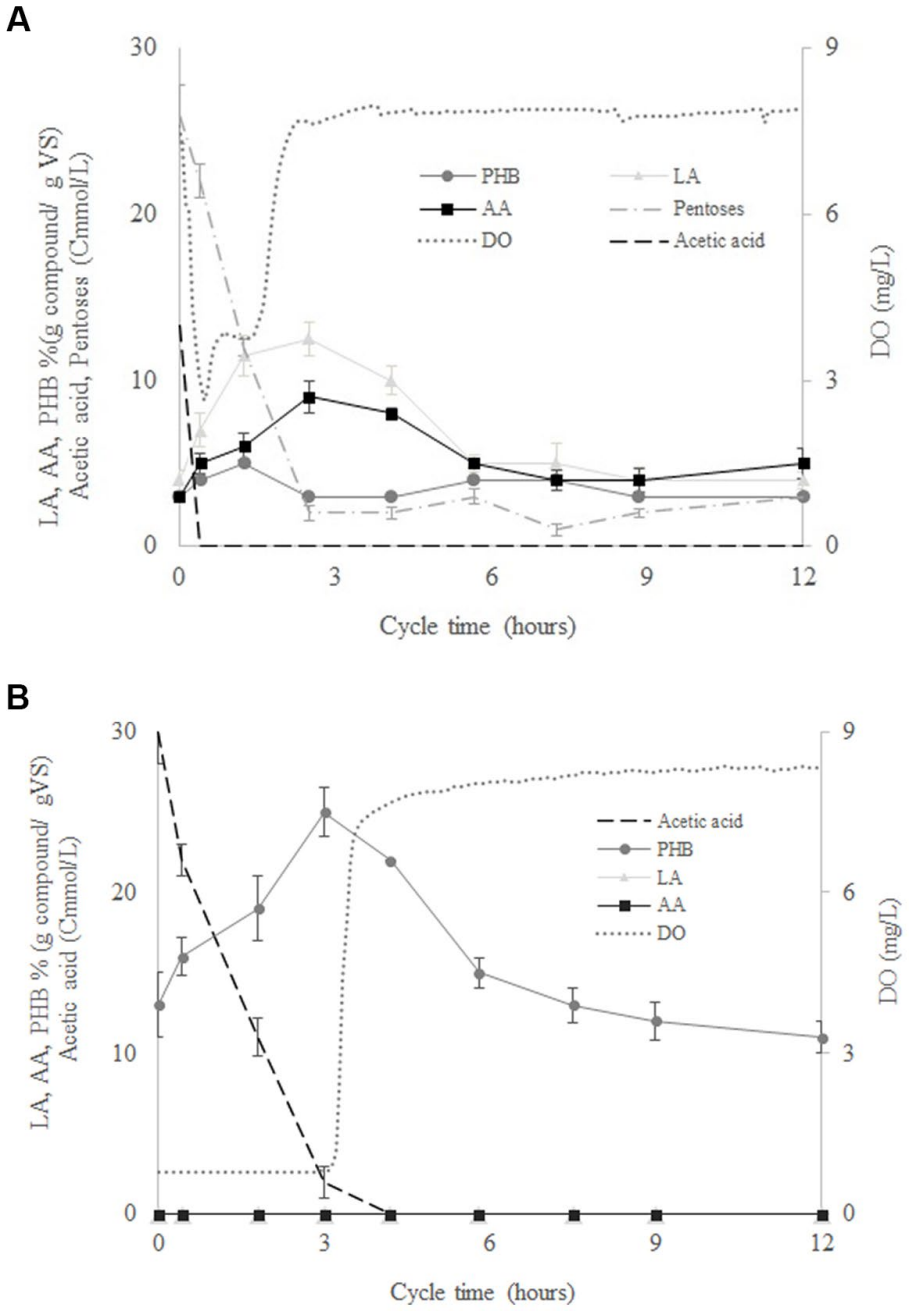
Variation of the concentration of levulinic acid (LA), adipic acid (AA), polyhydroxybutyrate (PHB), pentoses, acetate, volatile solids (*VS*), and dissolved oxygen (DO) throughout the cycle duration for SBR1 **(A)** and SBR2 **(B)**.

Figure 2A shows the variation of the mean concentrations of LA, AA, PHB, acetate and pentoses determined for four culture cycles performed by SBR1 during operation days 40, 42, 44, and 50. According to the variation in the concentration of the pentoses, SHH was consumed in around 2.5 h after the start of the cycle. At the same time, after a rapid drop from 8 to 2.8 mg/L, the dissolved oxygen increased again to a value of 8 mg/L (Figure 2A). This indicates the end of the feast phase and the beginning of the famine phase. The intracellular AA content increased from 3 to 9% (g compound/g *VS*) in the feast phase and decreased from 9% to 5% (g compound/g *VS*) in the famine phase. LA increased from 4% to 13% in the feast phase and decreased from 13 to 4% in the famine phase. Meanwhile, PHB remained around 3% (g compound/g *VS*) during the whole cycle (Figure 2A). The results showed that during the famine phase, the MMC were able to consume the AA and LA accumulated during the feast phase, probably due to external substrate limitation. Thus, AA and LA could be considered another carbon reserve like PHB. Figure 2B shows the variation of the mean concentrations of PHB, DO and acetate measured in four operational cycles performed in SBR2, at operation days 40, 42, 44, and 50. DO concentration increased from 1 g/L to 8 g/L at the end of the feast phase (after around 3 h of operation), when PHB production reached a concentration up to 25% (g compound/g *VS*). However, the PHB concentration decreased during the famine phase to values around 11% (g compound/g *VS*). AA and LA production were negligible throughout the cycle duration (Figure 2B). These results confirmed that the AA and LA production benefitted from the feeding of a complex substrate such as SHH to the MMC.

To date, to the best of our knowledge, the biological synthesis of AA has been only described from glucose and for metabolically engineered cultures of *Saccharomyces cerevisiae* (Zhang et al., 2020) or *Escherichia coli* (Zhou et al., 2020). AA biosynthesis using MMC feed with SHH has not been reported previously. LA biosynthesis using MMC feed with SHH has recently been reported by Pinto-Ibieta et al. (2020), where the LA concentration reached a value of up to 32% (g compound/g *VS*). The present research demonstrated that not only genetically modified microorganisms can metabolise pentoses into AA or LA. On the other hand, some studies have achieved an accumulation of PHB of up to 20% (g compound/g *VS*) using synthetic pentoses or lignocellulose hydrolysate to feed MMC under a F/F strategy (Zhang et al., 2016; Yin et al., 2019b). The differences in the profile of the accumulated compounds could be explained by the microbial composition of the selected MMC, as the microbial compositions in those studies differed from that used in the present research. In this sense, Oliveira et al. (2017) reported that the selected MMC will be different as a consequence of changes in the feed strategy. The different operational conditions established for the F/F culture strategy in this work versus those established by Zhang et al., 2016 will probably lead to a MMC enriched in LA-and AA-producing microorganisms.

Production of LA, but not of AA, production was achieved in our previous studies using SHH (Pinto-Ibieta et al., 2020). Table 2 shows a comparative summary of the results and operational conditions reported by Pinto-Ibieta et al. (2020) and those for SBR1 in the present work. In both cases, SHH was the carbon source, but the operational conditions of the F/F culture strategy were different, i.e., different DO, organic loading rate and nutrient ratio were fixed (Table 2). Therefore, the type of compound (LA and AA) produced could be a response of microorganisms to these differences in the established operational conditions. For instance, Table 2 shows that when DO concentration during the feast phase was higher than 3 mg/L, the selected MMC showed a tendency to synthesize AA in co-production with LA. By contrast, when the DO concentration was lower than 3 mg/L, the microorganisms synthesised only LA. Bart and Cavallaro (2015) reported that depending on the metabolic pathway type through which AA is synthesised using pure cultures, anaerobic systems could be more efficient than aerobic, or *vice-versa*. On the other hand, metabolic pathways to synthesise succinyl-CoA (intermediary in AA synthesis) were reported as being more efficient in the microaerobic system than aerobic or anaerobic. Therefore, DO concentration would be a crucial operational parameter to be controlled to favour the metabolic pathways related to AA production. This is also consistent with Pinto-Ibieta et al. (2021), where different operational conditions (DO, F/F ratio, SBR cycle length, organic loading rate (OLR), pH, C/N, and temperature) established for the F/F culture strategy were compiled and analysed, showing how the variation of these operational conditions favoured the accumulation of some compounds over others. These authors found that DO concentration was the variable that most clearly influenced the accumulation of targeted compounds. At DO concentrations higher than 3 mg/L, MMC tended to synthesise PG and TAG, while DO concentrations lower than 3 mg/L favoured the production of PHB and LA. Finally, the greater accumulation of LA observed in Pinto-Ibieta et al. (2020) than in the present study may be due to the lower OLR used in the present work. According to de Oliveira et al. (2019) the amount of PHB produced will increase as the organic loading rate increases, as long as the organic loading rate does not result in substrate inhibition.

**Table 2:** Levulinic acid (LA), adipic acid (AA), and polyhydroxybutyrate (PHB) production obtained per operation cycle by F/F culture strategy under different operational conditions, using SHH as carbon source per one operation cycle.

	LA % (g compound/g *VS*)	AA % (g compound/g *VS*)	PHB % (g compound/g *VS*)	DO during feast phase (mg/L)	C/N/P	OLR (mMC/d)
SBR fed with SHH reported in our previous work (Pinto-Ibieta et al., 2020)	37	0	3	< 3	45/2/1	90
SBR1 fed with SHH reported in present work	13	9	3	> 3	30/0.9/0.1	60

DO, dissolved oxygen; OLR, organic loading rate; SHH, synthetic hemicellulose hydrolysate; SBR, sequential batch reactor; *VS*, volatile solids.

Although the LA and AA co-production was possible in the present work, further studies with MMC are required to determine which of these variables could be the principal factor that favours synthesis and control in the selective accumulation of both AA and LA. In particular, the effect of DO concentration on the F/F culture strategy should be studied, as this seems to be the main variable that affects the synthesis of AA over LA. Nevertheless, the results indicate that under the operational conditions evaluated during the F/F culture strategy, the adapted MMC contain microorganisms with the required enzymatic pool for bioconversion of pentoses into AA and LA.

### 3.2. Batch assays for improvement of LA, AA, and PHB accumulation by previously selected microorganisms in SBR reactors

**Figure 3:**
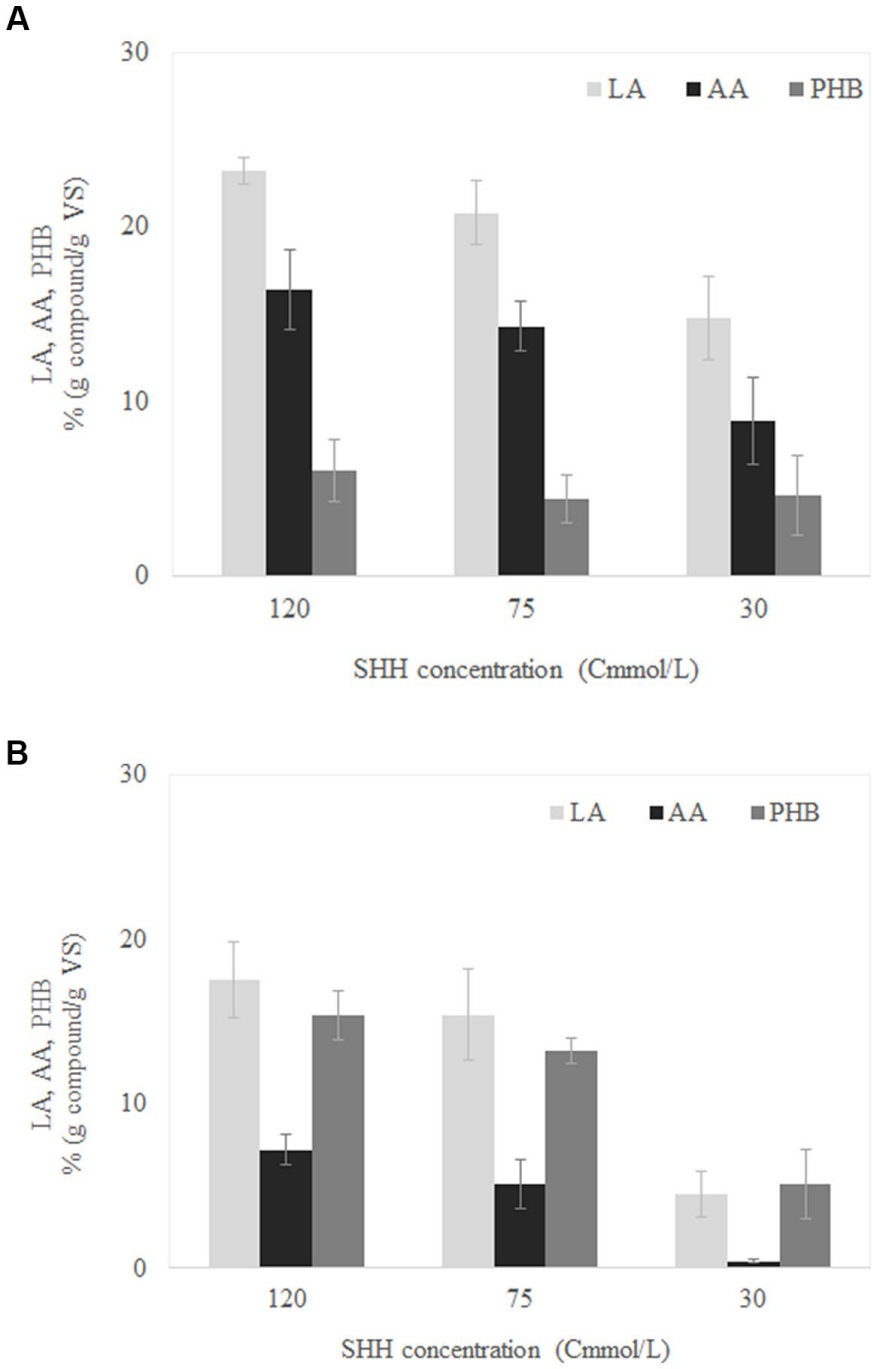
Maximum levulinic acid (LA), adipic acid (AA), polyhydroxybutyrate (PHB) accumulation capacity for mixed microbial cultures adapted in SBR1 **(A)** and SBR2 **(B)** at different synthetic hemicellulose hydrolysate (SHH) concentrations.

Figure 3A shows the concentrations of LA, AA, and PHB reached for the different SHH concentrations evaluated in the batch experiments conducted with the MMC previously selected in SBR1. For the three SHH concentrations evaluated, it was possible to accumulate the three added-value compounds identified in SBR1: LA, AA, and PHB. The highest concentration of SHH (120 Cmmol/L) produced the highest accumulation of each compound, obtaining yields of 23 ± 1, 16 ± 2, and 6 ± 2% g compound/g *VS* of LA, AA, and PHB, respectively (Figure 3A). When using 75 Cmmol/L, the LA, AA, and PHB accumulation concentrations were 20 ± 2, 14 ± 1, and 4 ± 1% (g compound/g *VS*), respectively; no significative difference from the latter was found in the values obtained at 120 Cmmol/L (Supplementary Table S1). The higher concentrations observed at 120 Cmmol/L could indicate that SHH was not inhibitory at this concentration (de Oliveira et al., 2019). The lowest accumulations were obtained at 30 Cmmol/L, with yields of only 15 ± 3, 9 ± 2, and 5 ± 2% (g compound/g *VS*) for LA, AA and PHB, respectively (Figure 3A), values which showed a significative reduction compared to those obtained at 120 Cmmol/L (Supplementary Table S1). This decrease could be caused by a limitation in the substrate, which could leading to a microbial growth limitation (Alburqueque et al., 2010; Pinto-Ibieta et al., 2021).

The use of MMC from SBR2 instead of SBR1 in the batch experiments resulted in general in a higher concentration of PHB and lower concentration of AA and LA compared to the results obtained using SBR1 (Figure 3). Specifically, by feeding with SHH at 120 Cmmol/L, it was possible to accumulate LA, AA, and PHB at concentrations of 18 ± 2, 7 ± 1, and 15 ± 2% (g compound/g *VS*), respectively (Figure 3B). On the other hand, at 30 Cmmol/L, the production of both LA and PHB was 5% (g compound/g *VS*), while no production of AA was observed. As in the reactors inoculated with the MMC obtained from SBR1, the statistical analysis (ANOVA) for the reactors inoculated with the MMC obtained from SBR2 only showed a significant difference when comparing 120 versus 30 Cmmol/L (Supplementary Table S2).

These results indicate that the simultaneous production of LA, AA, and PHB was possible using both adaptations of MMC under batch conditions (from SBR1 and SBR2), although the long-term adaptation of the MMC to the SHH in SBR1 favoured the accumulation of AA and LA. It may be noted that the origin and adaptation of the MMC could be a key point for the accumulation of high added-value compounds, as it is indicated by the statistical differences observed in the accumulation extents obtained from the MMC from SBR1 and SBR2 (Supplementary Table S3).

**Figure 4:**
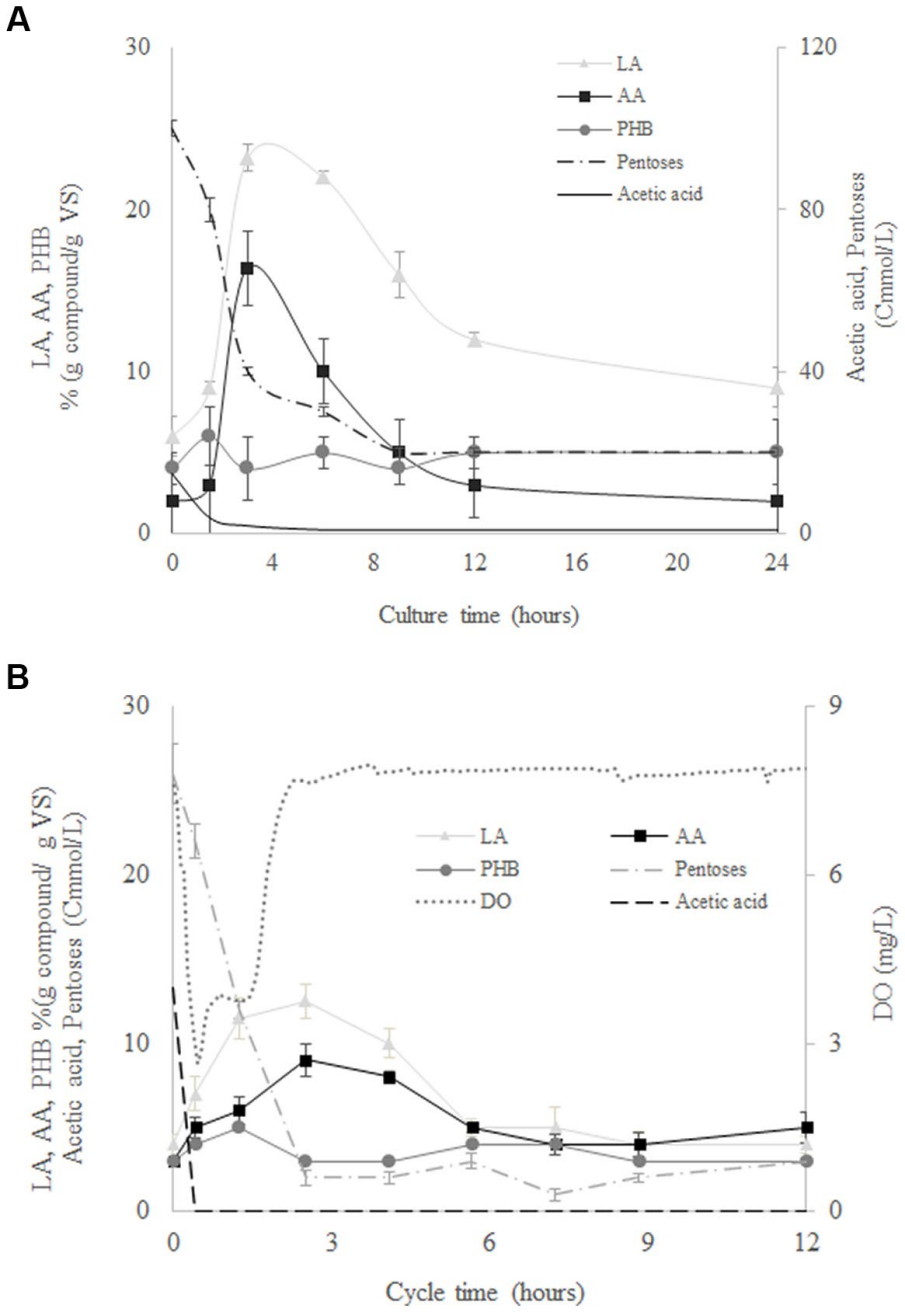
Evolution of levulinic acid (LA), adipic acid (AA), polyhydroxybutyrate (PHB), pentoses, acetate, and volatile solids (*VS*) during batch culture at 120 Cmmol/L for mixed microbial cultures from SBR1 **(A)** and SBR2 **(B)**.

Figure 4 shows the evolution of pentoses, biomass, LA, AA, and PHB during the culture time for the assays at 120 Cmmol/L using MMC from SBR1 (Figure 4B) and from SBR2 (Figure 4B) as the inoculum. Both reactors resulted in similar production and composition of the accumulated compounds despite of the different MMC used (Figure 4). Pentoses and acetic acid were rapidly consumed while AA, LA, and PHB were produced at the same time. The maximum accumulation of AA and LA was reached at the time when the pentoses were exhausted (around 3 h). Likewise, the maximum PHB was reached once the acetic acid was exhausted in the reactors (Figure 4). Similar behaviours were observed for AA, LA, and PHB, with concentrations decreasing after maximum generation. These results could indicate AA and LA storage in some kind of carbon source similar to PHB. Therefore, MMC adapted from SBR2 were not able to synthesise LA and AA using acetate, but the same microorganisms fed with SHH were capable of synthesising LA and AA, confirming that the production of AA and LA depends on the use of SHH as carbon source. This was expected since the metabolic pathways described for both AA and LA production begin with hexoses and/or pentoses as substrate (Lee et al., 2019).

Although both MMC types were able to synthesise LA, AA, and PHB when cultivated in batch conditions, the culture yields were different. As expected, MMC adapted in SBR1 with SHH were able to produce higher yields of AA and LA than those from SBR2. On the other hand, the co-production of LA and PHB was favoured in the batch assays conducted with MMC selected in SBR2. Therefore, the kind of substrate used in the selection stage could stimulate the production of specific compounds in a subsequent batch stage. The possible benefits of this co-production should be evaluated considering the yields of each compound, their final properties, the difficulties, and costs of separation, etc. In addition, it is important to identify the microbial communities and their metabolic pathway involved in AA and LA synthesis.

## 4. Conclusion

This research demonstrate that it is possible to accumulate AA from MMC, in co-production with LA, using SHH as a non-fermented carbon source and selecting the right operational conditions of the F/F culture strategy. Depending on the carbon source used during the adaptation stage (SHH or acetic acid), different proportions of AA, LA, and PHB were obtained during the accumulation stage in the batch assays. Our study indicates that MMC have the versatility to produce different added-value compounds from SHH, demonstrating that the production of one compound or another can be favoured depending on the operational conditions established, especially DO concentration. Nevertheless, further research is needed into the optimising the operational conditions of the F/F culture strategy to improve the AA and LA production yields. Furthermore, the adapted microbial community must be identified to understand the intracellular mechanisms for AA and LA accumulation.

## Data availability statement

The original contributions presented in the study are included in the article/Supplementary material, further inquiries can be directed to the corresponding author.

## Author contributions

FP-I, MC, GC, and FC contributed to conception and design of the study. FP-I organized the database and wrote the first draft of the manuscript. MC, GC, FC, and AS provided guidance and suggestions for the experimental design and discussed the results. FP-I, MC, FC, GC, FF, MA, and AS wrote and edited the manuscript. All authors contributed to the article and approved the submitted version.

## Funding

This research was funded by Fondecyt Postdoctorado No. 3210626, Agencia Nacional de Investigación y Desarrollo de Chile, ANID.

## Conflict of interest

The authors declare that the research was conducted in the absence of any commercial or financial relationships that could be construed as a potential conflict of interest.

## Publisher’s note

All claims expressed in this article are solely those of the authors and do not necessarily represent those of their affiliated organizations, or those of the publisher, the editors and the reviewers. Any product that may be evaluated in this article, or claim that may be made by its manufacturer, is not guaranteed or endorsed by the publisher.
