# Supplementary material for: Toward the use of mixed microbial cultures for the biological production of adipic and levulinic acid
Source: Front Microbiol. 2023 Jun 28;14:1224543. doi: 10.3389/fmicb.2023.1224543 (PMC10338001; doi:10.3389/fmicb.2023.1224543)
Supplement: Supplementary file 1 [file Table_1.docx]

| **ANOVA comparison between all concentration studied for LA production in reactors inoculated with MMC obtained from SBR1** | | | | | | | |
| --- | --- | --- | --- | --- | --- | --- | --- |
| *Source of Variation* | *SS* | *df* | *MS* | ***F*** | *P-value* | ***F crit*** | Statistical difference? |
| Between Groups | 102 | 2 | 50,85 | **7,336** | 0,024 | **5,143** | yes |
| Within Groups | 42 | 6 | 6,93 |  |  |  |  |
| Total | 143 | 8 |  |  |  |  |  |
| **ANOVA comparison between LA production using 120 and 75 Cmmol/L of added carbon source in reactors inoculated with MMC obtained from SBR1** | | | | | | | |
| *Source of Variation* | *SS* | *df* | *MS* | ***F*** | *P-value* | ***F crit*** | Statistical difference? |
| Between Groups | 8 | 1 | 8,17 | **2,808** | 0,169 | **7,709** | No |
| Within Groups | 12 | 4 | 2,91 |  |  |  |  |
| Total | 20 | 5 |  |  |  |  |  |
| **ANOVA comparison between LA production using 120 and 30 Cmmol/L of added carbon source in reactors inoculated with MMC obtained from SBR1** | | | | | | | |
| *Source of Variation* | *SS* | *df* | *MS* | ***F*** | *P-value* | ***F crit*** | Statistical difference? |
| Between Groups | 98 | 1 | 98,42 | **15,227** | 0,018 | **7,709** | yes |
| Within Groups | 26 | 4 | 6,46 |  |  |  |  |
| Total | 124 | 5 |  |  |  |  |  |
| **ANOVA comparison between all concentration studied for AA production in reactors inoculated with MMC obtained from SBR1** | | | | | | | |
| *Source of Variation* | *SS* | *df* | *MS* | ***F*** | *P-value* | ***F crit*** | Statistical difference? |
| Between Groups | 97 | 2 | 48,54 | **14,611** | 0,005 | **5,143** | yes |
| Within Groups | 20 | 6 | 3,32 |  |  |  |  |
| Total | 117 | 8 |  |  |  |  |  |
| **ANOVA comparison between AA production using 120 and 75 Cmmol/L of added carbon source in reactors inoculated with MMC obtained from SBR1** | | | | | | | |
| *Source of Variation* | *SS* | *df* | *MS* | ***F*** | *P-value* | ***F crit*** | Statistical difference? |
| Between Groups | 6 | 1 | 6,41 | **1,920** | 0,238 | **7,709** | No |
| Within Groups | 13 | 4 | 3,34 |  |  |  |  |
| Total | 20 | 5 |  |  |  |  |  |
| **ANOVA comparison between AA production using 120 and 30 Cmmol/L of added carbon source in reactors inoculated with MMC obtained from SBR1** | | | | | | | |
| *Source of Variation* | *SS* | *df* | *MS* | ***F*** | *P-value* | ***F crit*** | Statistical difference? |
| Between Groups | 90 | 1 | 90,48 | 22,332 | 0,009 | 7,709 | yes |
| Within Groups | 16 | 4 | 4,05 |  |  |  |  |
| Total | 107 | 5 |  |  |  |  |  |

S.1. ANOVA for statistical analysis of data from figure 3.a and 3.b.

Table S.1. Comparison between LA and AA productions at the different concentrations studied, for the inoculum obtained from SBR1.

Table S.2. Comparison between LA and AA production at the different concentrations studied, for the inoculum obtained from SBR2.

| **ANOVA comparison between all concentration studied for LA production in reactors inoculated with MMC obtained from SBR2** | | | | | | | |
| --- | --- | --- | --- | --- | --- | --- | --- |
| *Source of Variation* | *SS* | *df* | *MS* | ***F*** | *P-value* | ***F crit*** | Statistical difference? |
| Between Groups | 292,40 | 2 | 146,20 | 29,404 | 0,001 | 5,143 | yes |
| Within Groups | 29,83 | 6 | 4,97 |  |  |  |  |
| Total | 322,24 | 8 |  |  |  |  |  |
| **ANOVA comparison between LA production using 120 and 75 Cmmol/L of added carbon source in reactors inoculated with MMC obtained from SBR2** | | | | | | | |
| *Source of Variation* | *SS* | *df* | *MS* | ***F*** | *P-value* | ***F crit*** | Statistical difference? |
| Between Groups | 352 | 1 | 352,5 | 0,199 | 0,671 | 5,987 | No |
| Within Groups | 10634 | 6 | 1772,3 |  |  |  |  |
| Total | 10986 | 7 |  |  |  |  |  |
| **ANOVA comparison between LA production using 120 and 30 Cmmol/L of carbon source in reactors inoculated with MMC obtained from SBR2** | | | | | | | |
| *Source of Variation* | *SS* | *df* | *MS* | ***F*** | *P-value* | ***F crit*** | Statistical difference? |
| Between Groups | 252 | 1 | 252,2 | 69,541 | 0,001 | 7,709 | Yes |
| Within Groups | 15 | 4 | 3,6 |  |  |  |  |
| Total | 267 | 5 |  |  |  |  |  |
| **ANOVA comparison between all concentration studied for AA production in reactors inoculated with MMC obtained from SBR2** | | | | | | | |
| *Source of Variation* | *SS* | *df* | *MS* | ***F*** | *P-value* | ***F crit*** | Statistical difference? |
| Between Groups | 73 | 2 | 36,37 | 35,541 | 0,001 | 5,143 | yes |
| Within Groups | 6 | 6 | 1,02 |  |  |  |  |
| Total | 79 | 8 |  |  |  |  |  |
| **ANOVA comparison between AA production using 120 and 75 Cmmol/L of carbon source in reactors inoculated with MMC obtained from SBR2** | | | | | | | |
| *Source of Variation* | *SS* | *df* | *MS* | ***F*** | *P-value* | ***F crit*** | Statistical difference? |
| Between Groups | 7 | 1 | 6,62 | 4,324 | 0,106 | 7,709 | No |
| Within Groups | 6 | 4 | 1,53 |  |  |  |  |
| Total | 13 | 5 |  |  |  |  |  |
| **ANOVA comparison between AA production using 120 and 30 Cmmol/L of carbon source in reactors inoculated with MMC obtained from SBR2** | | | | | | | |
| *Source of Variation* | *SS* | *df* | *MS* | ***F*** | *P-value* | ***F crit*** | Statistical difference? |
| Between Groups | 69 | 1 | 69,36 | **169,171** | 0,001 | **7,709** | yes |
| Within Groups | 2 | 4 | 0,41 |  |  |  |  |
| Total | 71 | 5 |  |  |  |  |  |

Table S.3. comparison between the reactors inoculated with MMC from SBR1 and SBR2 specifically comparison between LA and AA productions obtained for each concentration studied.

| **ANOVA comparison between reactores considering LA production at 120 Cmmol/L** | | | | | | | |
| --- | --- | --- | --- | --- | --- | --- | --- |
| *Source of Variation* | *SS* | *df* | *MS* | ***F*** | *P-value* | ***F crit*** | Statistical difference? |
| Between Groups | 49 | 1 | 48,74 | 15,480 | 0,017 | 7,709 | yes |
| Within Groups | 13 | 4 | 3,15 |  |  |  |  |
| Total | 61 | 5 |  |  |  |  |  |
| **ANOVA comparison between reactores considering AA production at 120 Cmmol/L** | | | | | | | |
| *Source of Variation* | *SS* | *df* | *MS* | ***F*** | *P-value* | ***F crit*** | Statistical difference? |
| Between Groups | 127 | 1 | 126,96 | 45,181 | 0,003 | 7,709 | yes |
| Within Groups | 11 | 4 | 2,81 |  |  |  |  |
| Total | 138 | 5 |  |  |  |  |  |
| **ANOVA comparison between considering LA production at 75 Cmmol/L** | | | | | | | |
| *Source of Variation* | *SS* | *df* | *MS* | ***F*** | *P-value* | ***F crit*** | Statistical difference? |
| Between Groups | 55 | 1 | 55,21 | 8,248 | 0,045 | 7,709 | yes |
| Within Groups | 27 | 4 | 6,69 |  |  |  |  |
| Total | 82 | 5 |  |  |  |  |  |
| **ANOVA comparison between reactores considering AA production at 75 Cmmol/L** | | | | | | | |
| *Source of Variation* | *SS* | *df* | *MS* | ***F*** | *P-value* | ***F crit*** | Statistical difference? |
| Between Groups | 128 | 1 | 127,88 | 62,179 | 0,001 | 7,709 | yes |
| Within Groups | 8 | 4 | 2,06 |  |  |  |  |
| Total | 136 | 5 |  |  |  |  |  |
| **ANOVA comparison between reactores considering LA production at 30 Cmmol/L** | | | | | | | |
| *Source of Variation* | *SS* | *df* | *MS* | ***F*** | *P-value* | ***F crit*** | Statistical difference? |
| Between Groups | 167 | 1 | 167,48 | 24,127 | 0,008 | 7,709 | yes |
| Within Groups | 28 | 4 | 6,94 |  |  |  |  |
| Total | 195 | 5 |  |  |  |  |  |
| **ANOVA comparison between reactores considering AA production at 30 Cmmol/L** | | | | | | | |
| *Source of Variation* | *SS* | *df* | *MS* | ***F*** | *P-value* | ***F crit*** | Statistical difference? |
| Between Groups | 102 | 1 | 101,68 | 61,563 | 0,001 | 7,709 | yes |
| Within Groups | 7 | 4 | 1,65 |  |  |  |  |
| Total | 108 | 5 |  |  |  |  |  |
