# Supplementary material for: Toward the use of mixed microbial cultures for the biological production of adipic and levulinic acid
Source: Front Microbiol. 2023 Jun 28;14:1224543. doi: 10.3389/fmicb.2023.1224543 (PMC10338001; doi:10.3389/fmicb.2023.1224543)
Supplement: Supplementary file 2 [file Data_Sheet_1.docx]

***Figure Captions***

**Figure 1.** Evolution of levulinic acid (LA), adipic acid (AA), polyhydroxybutyrate (PHB), and volatile solids (VS) in the 50 days performed by SBR1 (a) and SBR2 (b).

**Figure 2.** Variation of the concentration of levulinic acid (LA), adipic acid (AA), polyhydroxybutyrate (PHB), pentoses, acetate, volatile solids (VS), and dissolved oxygen (DO) throughout the cycle duration for SBR1 (a) and SBR2 (b).

**Figure 3.** Maximum levulinic acid (LA), adipic acid (AA), polyhydroxybutyrate (PHB) accumulation capacity for mixed microbial cultures adapted in SBR1 (a) and SBR2 (b) at different synthetic hemicellulose hydrolysate (SHH) concentrations.

**Figure 4.** Evolution of levulinic acid (LA), adipic acid (AA), polyhydroxybutyrate (PHB), pentoses, acetate, and volatile solids (VS) during batch culture at 120 Cmmol/L for mixed microbial cultures from SBR1 (a) and SBR2 (b).

1.a


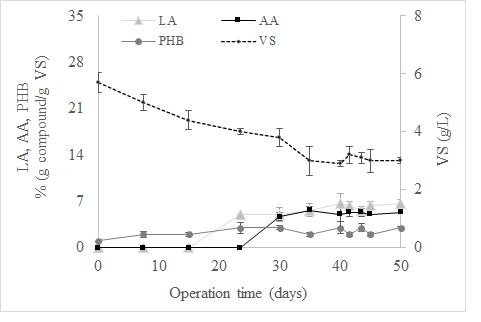


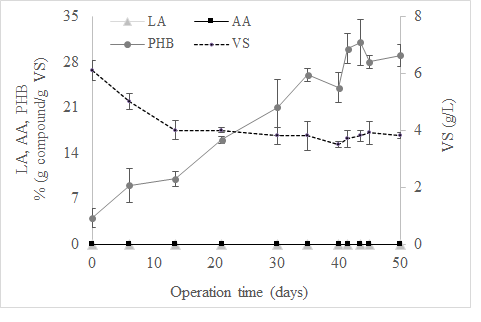
1.b

2.a


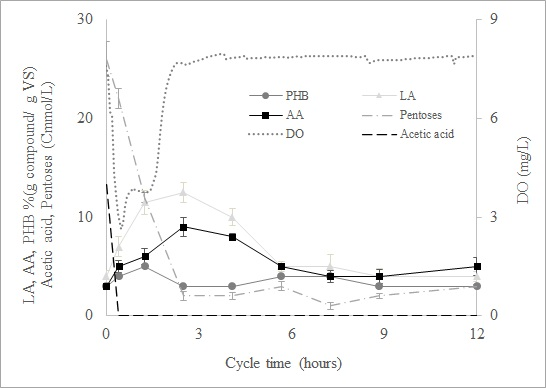


2.b


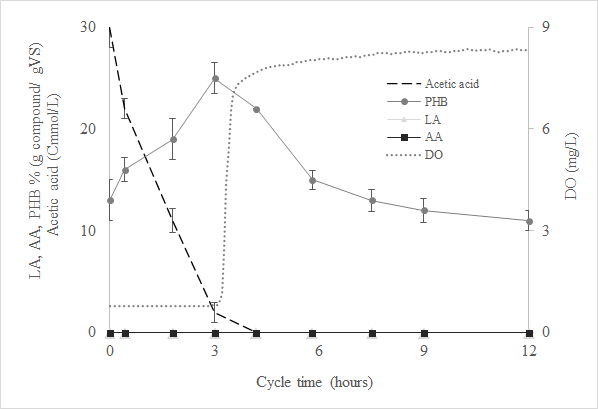


3.a


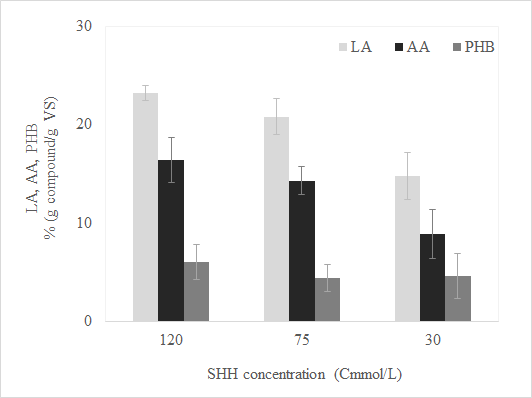


3.b


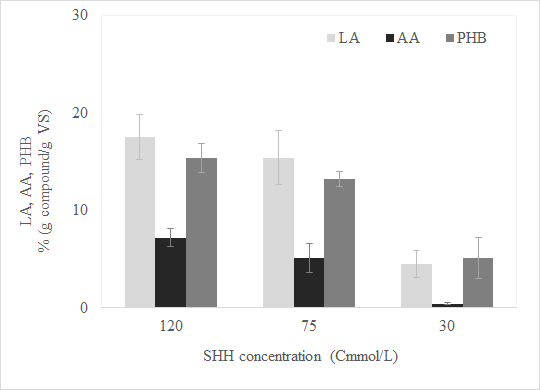


4.a


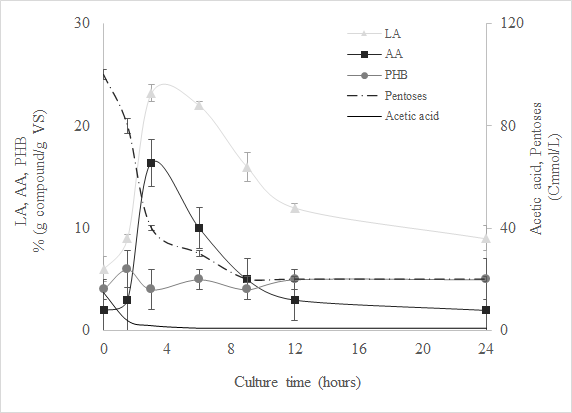


4.b
